# Density-dependent sex ratio and sex-specific preference for host traits in parasitic bat flies

**DOI:** 10.1186/s13071-017-2340-0

**Published:** 2017-08-29

**Authors:** Tamara Szentiványi, Orsolya Vincze, Péter Estók

**Affiliations:** 1 Department of Evolutionary Zoology and Human Biology, University of Debrecen, Egyetem tér 1, H-4032, Debrecen, H-4032 Hungary; 2 Department of Ecology and Evolution, University of Lausanne, Biophore, CH-1015 Lausanne, Switzerland; 3 Museum of Zoology, Palais de Rumine, Place de la Riponne 6, CH-1014 Lausanne, Switzerland; 4 Department of Evolutionary Zoology and Human Biology, MTA-DE “Lendület” Behavioural Ecology Research Group, University of Debrecen, Debrecen, H-4032 Hungary; 5 Hungarian Department of Biology and Ecology, Evolutionary Ecology Group, Babeş-Bolyai University, RO-400006 Cluj-Napoca, Romania; 6 Department of Zoology, Eszterházy Károly University, Eger, H-3300 Hungary

**Keywords:** Bat fly, Chiroptera, Density-dependence, Parasite intensity, Sex ratio

## Abstract

**Background:**

Deviation of sex ratios from unity in wild animal populations has recently been demonstrated to be far more prevalent than previously thought. Ectoparasites are prominent examples of this bias, given that their sex ratios vary from strongly female- to strongly male-biased both among hosts and at the metapopulation level. To date our knowledge is very limited on how and why these biased sex ratios develop. It was suggested that sex ratio and sex-specific aggregation of ectoparasites might be shaped by the ecology, behaviour and physiology of both hosts and their parasites. Here we investigate a highly specialised, hematophagous bat fly species with strong potential to move between hosts, arguably limited inbreeding effects, off-host developmental stages and extended parental care.

**Results:**

We collected a total of 796 *Nycteribia kolenatii* bat flies from 147 individual bats using fumigation and subsequently determined their sex. We report a balanced sex ratio at the metapopulation level and a highly variable sex ratio among infrapopulations ranging from 100% male to 100% female. We show that infrapopulation sex ratio is not random and is highly correlated with infrapopulation size. Sex ratio is highly male biased in small and highly female biased in large infrapopulations. We show that this pattern is most probably the result of sex-specific preference in bat flies for host traits, most likely combined with a higher mobility of males. We demonstrate that female bat flies exert a strong preference for high host body condition and female hosts, while the distribution of males is more even.

**Conclusions:**

Our results suggest that locally biased sex ratios can develop due to sex-specific habitat preference of parasites. Moreover, it is apparent that the sex of both hosts and parasites need to be accounted for when a better understanding of host-parasite systems is targeted.

**Electronic supplementary material:**

The online version of this article (doi:10.1186/s13071-017-2340-0) contains supplementary material, which is available to authorized users.

## Background

Host-parasite interactions are of key importance in evolutionary biology, defining coevolutionary arms races, demography and fitness of both hosts and parasites [1]. On the one hand, the reproductive output of hosts largely depends on their ability to avoid or mitigate parasite burden that require various adaptations at the level of behaviour and physiology. These adaptations often differ between host individuals and large part of this within-species variance can be attributed to different life-history strategies of male and female hosts [2, 3]. On the other hand, parasites target the most efficient exploitation of their hosts and maximisation of their own reproductive output. Nonetheless, sexual differences are often observed in parasite species including dimorphism in size, mobility, behaviour, competitive ability, or even genetic structure [4–6], likely to result in sex-specific fitness maximisation strategies, similarly to their hosts. Accounting for sex differences in hosts and their parasites is therefore of equal importance when a better understanding of host-parasite systems is targeted.

Some of the most intriguing current conundrums of parasitology appear to be closely linked to parasite sex. For example, biased sex ratios are common in ectoparasites and despite strong scientific attention, driving causes of sex ratio distortions remain controversial in this group [5, 7, 8]. The most common sex ratio in ectoparasites is female-biased, but equal and strongly male-biased sex ratios are also commonly encountered both on a species level and within species, among different host individuals [5, 7]. It remains to be explored why sex ratios of parasite populations vary so widely and what adaptive significance do sex ratio fluctuations have [6].

Bats represent widely used model organisms for parasitological studies and they are hosts to a high diversity of parasites. Among ectoparasites, four insect orders have been recorded on bats, including bat flies (Diptera), fleas (Siphonaptera), true bugs (Hemiptera) and earwigs (Dermaptera) [1]. Bat flies are present with two families, Nycteribiidae and Streblidae, with 16 species of nycteribiids and only one streblid species recorded in Europe to date [9]. Nycteribiids are obligate, wingless, highly specialised blood-feeding ectoparasites of bats. They reproduce by adenotrophic viviparity; females produce a single offspring at a time, the egg and then the larva develops, feeds, moults within the uterus and consumes the secretion of special “milk glands”. Pregnant females leave their hosts to larviposit the third-instar larvae on the walls of the host’s roosting sites. The deposited larva pupates immediately and adult flies usually emerge within 3 to 4 weeks following pupation [1]. This highly specialised host-parasite system provides an exceptional opportunity to study host-parasite interactions due to its rare properties. Bats roost communally, with high frequency of prolonged physical contact among dozens to hundreds of individuals, providing ectoparasites with the possibility of free movement between hosts and to perform active choice of hosts. Moreover, off-host developmental stages and larviposition, the relatively long, off-host metamorphosis of the larvae, as well as the sequential roost site utilisation ensures genetic mixing of parasites among hosts. These characters largely reduce inbreeding, a factor that has been suggested to deeply influence demography and population dynamics of most parasite species.

Several hypotheses have been proposed to explain sex ratio variation in arthropod ectoparasites. These include biased prepartum sex ratio, local mate competition, sex differences in longevity, selective grooming or sampling bias [1, 5, 7, 10, 11]. Skewed sex ratios of bat flies at emergence has the potential to strongly influence adult sex ratios. Female-biased sex ratio at emergence was demonstrated in a streblid species, *Trichobius frequens*, but such sex ratio bias was not present among the adults in the same study population [8]. Moreover, Marshall [12] demonstrated that the prepartum sex ratio in the nycteribiid bat fly *Basilia hipsida* did not differ from unity. Biased prepartum sex ratio is predicted by the local mate competition (LMC) hypothesis [13]. In isolated populations, especially with limited male dispersal and considerable sibmating, parents and sons compete for mating opportunities. In such populations fitness returns of female offspring is higher than of males, therefore adaptive adjustment in offspring sex ratio occurs. The degree of inbreeding in bat flies is little known [5], but appears to be considerably low since chances of host switching is high. It is therefore unlikely that the LMC plays a significant role in shaping adult sex ratios of bat flies.

Sex-specific longevity is another parasite trait that is likely to bias adult sex ratios in ectoparasitic arthropods, toward an excess of the sex with the longer lifespan [7]. In nycteribiid bat flies sex-specific longevity has been documented before; average lifespan of males was 136 days, while that of females reached 195 days in *B. hipsida* [12]. Similarly, female-biased longevity was observed in a few closely related fly species [5]. Nonetheless, it is little known how general this sex-specific longevity is among different bat fly species and how significant its role is in shaping adult sex ratios. Other important factors, probably not independent of longevity, are sex-specific body size, mobility and detectability. Such differences might result in higher probability of a certain sex to be found or picked by the host or collected by human observers. These can result in sex-specific grooming or sampling bias, respectively. Similarly, sex-specific diurnal behaviour, especially concerning off-host behaviours, such as larviposition might contribute to sampling bias and erroneous sex ratio estimates [5, 8].

Besides fluctuation in their sex ratios bat flies exhibit considerable variance in their aggregations both at the intra- [14] and interspecific levels [15] even when hosts roost in close physical contact. Most host individuals harbour no or only a small number of parasites and only a few bear with higher parasite intensities (e.g. [16]). Infestation rates of hosts appear to be influenced by a wide range of behavioural, physiological and ecological traits of both hosts and their parasites. Among host traits, sex appears to play a particularly important role in this respect, partly due to sex-specific parasite exposure. In bats for instance females tend to roost in colonies, while males usually roost solitarily or in small aggregations [17–20]. This difference is often mentioned as a key factor responsible for the observed higher parasitism in female than in male bats. The close proximity of females might elicit the transmission and therefore offers very favourable circumstances for the parasites. Moreover, the presence of the juveniles at maternity colonies might also increase parasite reproductive output. Juveniles possess relatively weak immune systems and their antiparasitic behaviours are less elaborate than in adults, making this age class and associated females more prone to exploitation by parasites [21–23].

Sexual differences in parasitism is not always a result of host exposure. Some parasites actively switch to a certain host sex when given the opportunity [24]. Males in mammals, birds, reptiles as well as in invertebrates are usually more heavily parasitised than females [25–29]. Such sex differences in parasitism is often the result of the less competent immune systems of males compared to females [30], most commonly attributed to the pleiotropic effects of steroid reproductive hormones, e.g. testosterone [31]. Contrary to patterns observed in most vertebrate animals, in bats females appear to be subject to more intense parasitism than males [14, 24, 32–35] but see [23, 36]. In line with this, some of their parasites actively move to female hosts and their fitness is much higher on female than on male hosts (e.g. ectoparasitic mites in bats [24]). Such sexual differences might originate from physiological or behavioural trade-offs between self-maintenance (e.g. immunity, grooming) and other costly life-history components (e.g. mate attraction, pregnancy, lactation).

Another host trait suggested to influence the degree of parasitism is host body condition. It was for instance suggested that parasite should favor the host with the weakest immune system and therefore lowest body condition [22, 37]. On the other hand, positive association between the degree of parasitism and host body condition was also observed in numerous cases [22]. The association between host condition and degree of parasitism is controversial in bats too. Some studies found positive [38], some negative [39] and some no correlation between parasite numbers and host body condition [14, 34].

Within the framework of this study, we investigate a highly specialised host-parasite system of the Daubenton’s bats (*Myotis daubentonii*) and their obligate arthropod ectoparasite bat fly species *Nycteribia kolenatii*. Bat flies exhibit large variation in their sex ratios. In nycteribiid bat flies equal, female-biased and male-biased sex ratios were all frequently reported [5, 40, 41]. Our aim is to explore and discuss how sex-specific parasite traits might contribute to the development of skewed sex ratios in this group. In addition, we study sex ratio variation among hosts and the link between sex ratio and parasite density within hosts (i.e. infrapopulation size), host traits as well as sex-specific preference for host traits in bat flies.

## Methods

Fieldwork was carried out at several foraging sites of bats at a number of localities in Hungary, including Gemenc, Alba, Kislőd, Szentgál, Hajszabarna, Mátra, Abaliget, Vinye, Futómacskás, Makó, Bakony, Debrecen and Vízfő. Daubenton’s bats (*Myotis daubentonii*) were captured using mist nets set up in the proximity of caves and foraging sites. Captures took place from 1st April to 5th September 1999, but most individuals (72%) were captured in August. Body mass, forearm length and the sex of each bat was recorded and they were marked with an individually numbered aluminium ring. All ectoparasitic bat flies were collected from each bat using fumigation. The latter method has a wide range of benefits over traditional visual inspection. First, fumigation is a more thorough method of parasite collection and yields a very high proportion of parasites, contrary to traditional methods. Secondly, sampling is independent of the observer’s ability to find and capture often highly mobile parasites and is therefore less prone to bias [42]. Fumigation was carried out with a “Fair Isle Apparatus” developed for birds with slight modifications [42]. Bat flies were removed from the hosts and were stored at room temperature in plastic Eppendorf vials containing 70% ethanol. During subsequent examination the sex and species of each individual bat fly was determined under a stereomicroscope based on morphological differences described in Theodor [43]. Each bat was captured and examined for parasites only once. Sex and morphological measurements of a few individuals (i.e. 1 and 4, respectively) were not recorded, therefore sample sizes vary across the model, depending on explanatory variables included in these. Only data on parasitised individuals was recorded on the field, harbouring a minimum of one bat fly individual.

### Statistical analyses

To analyse sex ratio in parasitic bat flies we constructed generalised linear models (GLMs) with binomial error distribution. Each bat fly was handled as an individual data point, and their sex (F/M, coded with 0/1, respectively) was used as a dependent variable in the models. First, we tested whether sex ratio of the entire bat fly collection deviated from unity, by constructing an intercept model and testing the significance of the intercept term (Model 1). Secondly, to test whether sex ratio of bat flies varies among host individuals or whether they are sexually segregated, we added host identity as a fixed factor to Model 1 and tested its effect using likelihood ratio statistics (Model 2). Thirdly, to investigate how sex ratio of infrapopulations depends on host traits (sex, condition) and on infrapopulation size, by adding these explanatory variables and all possible second order interactions to Model 1 (Model 3). Non-significant interactions were removed from the models and their effects is not reported in the results. Host condition was calculated as scaled mass body condition [44, 45], i.e. residuals of a standard major axis (SMA) regression calculated between body mass and forearm length (original scale). Type II regression method was adopted, since SMA residuals were proved to be better indicators of energy reserves than traditional condition indices [44, 45]. SMA regression was constructed using the R package *lmodel2* [46].

Abundance of bat flies was analysed using generalised linear mixed models with Poisson error distribution. Number of bat flies on each host was included as the dependent variable, while host sex, host condition and the interaction between these two traits were used as explanatory variables. Abundance analyses were first run for total abundance, but were repeated for male and female bat fly abundances separately. Given that the degree of parasitism [12, 47] or sex ratio might vary seasonally [48] but see [5], we tested the effect of month on both bat fly densities and sex ratios. Note, however that month had no significant effect in any of the models, neither as main effect nor in interaction with other parameters, therefore this effect is not shown in the results. All statistical analyses were performed using R 3.3.2 (R Core Team 2016).

## Results

All bat flies collected from *Myotis daubentonii* bats belonged to a single bat fly species, *Nycteribia kolenatii*. A total of 796 bat flies (371 males, 425 females) were collected from 145 individual bats (84 males, 60 females). The number of bat flies collected from different host individuals (i.e. infrapopulation size) varied from 1 to 21 individuals (mean 5.49, SD 4.31) and their numbers exhibited Poisson distribution. Number of male and female bat flies collected from a single host individual was strongly positively correlated (Pearson’s product-moment correlation, *t* = 3.89, *df* = 143, *P* = 0.0002).

### Sex ratio and sexual segregation of parasites

Overall sex ratio of the collected bat flies was 0.47 (*n* = 796), indicating a slightly larger number of females compared to males. Note however that the intercept term in Model 1, did not reach significance, indicating that this ratio did not deviate significantly from unity (*Z* = -1.91, *P* = 0.0558). Host identity was a strong predictor of bat fly sex (Model 2, likelihood ratio statistics, *χ* ^2^ = 217.09, *df* = 144, *P* < 0.0001), indicating a strong variation in sex ratio across host individuals and sexual segregation of the parasites.

**Fig. 1:**
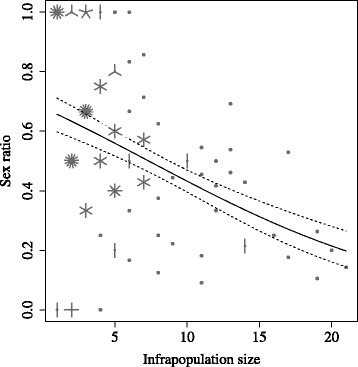
Sunflower plot showing the relationship between infrapopulation size and infrapopulation sex ratio on 145 hosts. Each point represents a different host individual. Number of overlapping data points are marked with increasing number of petals of the plotted points. Model predictions and associated 95% confidence intervals were obtained from a binomial GLM between bat fly sex and infrapopulation size

The results of the binomial GLMs showed that sex of the bat flies was independent of host body condition (Table 1). Moreover, host sex was a strong predictor of parasite sex (*t* = 3.59, *P* = 0.0003), although this effect disappeared when infrapopulation size was included in the model, indicating collinearity between host sex and infrapopulation size. Female hosts had a much higher probability to harbour female than male bat flies. Infrapopulation size was a strong predictor of bat fly sex (Fig. 1). Male bat flies were predominant in small infrapopulations (e.g. sex ratio was on average 0.86 in 14 infrapopulations consisting of a single individual) and was highly female biased in large infrapopulations (e.g. sex ratio was on average 0.17 in two infrapopulations with 20 and 21 bat flies each) (Fig. 1). Identical results were obtained when the sampling period was restricted to the mating season (August and September), excluding samples collected in the period between April and July.

**Table 1:** Results of a binomial GLM exploring variation in bat fly sex (0, female; 1, male) in relation to host sex, host condition and infrapopulation size. All second-order interactions have been tested but were removed due to their non-significant effects

	β (SE)	*t*	*P*
Intercept	0.58 (0.18)	3.19	0.0014
Host condition	0.02 (0.06)	0.35	0.7243
Host sex	0.25 (0.15)	1.6	0.1091
Infrapopulation size	-0.10 (0.02)	-6.33	< 0.0001
	*n* = 786		

*Abbreviation*: *SE* standard error

### Parasite abundance and host preference

**Fig. 2:**
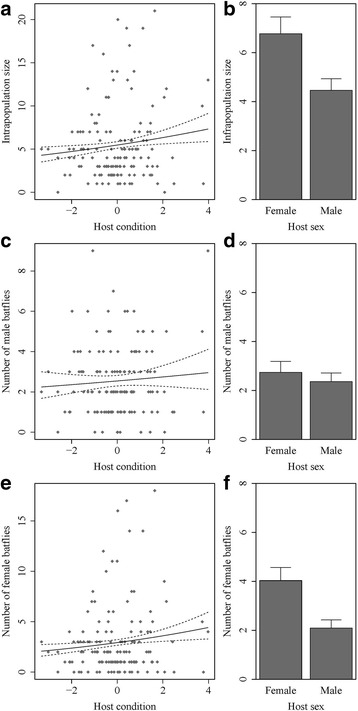
Relationship between host condition or sex and bat fly abundance for both sexes combined (**a, b**), as well as for males (**c, d**) and females (**e, f**) separately. Error lines and bars represent 95% confidence intervals. Slopes and means were obtained from single predictor GLMs between abundance and host condition or sex

Infrapopulation size of bat flies varied widely among hosts and was predicted by host sex and body condition (Fig. 2, Table 2). Infrapopulations were larger on hosts in better body condition and on female compared to male hosts. Note however, that sex-specific effects were detected when infrapopulation size was divided according to parasite sex and analysed separately. The number of male bat flies within hosts was influenced neither by host condition, nor by host sex. On the contrary, the number of female bat flies within hosts was correlated both with host condition and host sex. The number of female bat flies within hosts increased with increasing host body condition and was significantly larger in female than in male bat hosts.

**Table 2:** Results of GLMs investigavting variance in bat fly abundances across hosts in relation to host condition and sex. Abundance of all, and male and female bat flies were analysed. Non-significant second order interactions have been removed from all three models due to non-significance. All three models are based on 143 hosts and a total of 786 bat flies, 364 males and 422 females

	Total abundance			Male abundance			Female abundance		
	β (SE)	*t*	*P*	β (SE)	*t*	*P*	β (SE)	*t*	*P*
Intercept	1.92 (0.05)	38.82	< 0.0001	1.01 (0.08)	13.02	< 0.0001	1.41 (0.06)	21.98	< 0.0001
Host condition	0.12 (0.04)	2.89	0.0039	0.04 (0.04)	1.00	0.3161	0.12 (0.04)	3.20	0.0014
Host sex	-0.42 (0.07)	-5.82	< 0.0001	-0.14 (0.11)	-1.36	0.1735	-0.67 (0.1)	-6.78	< 0.0001
	*n* = 143			*n* = 143			*n* = 143		

*Abbreviation*: *SE* standard error

## Discussion

Here we explored a highly specialised host-ectoparasite system investigating sex ratio, aggregation and host preference of parasitic bat flies. Using an extensive sample size of both host and their parasites, collected using thorough and objective methodology we reached three important conclusions. First, we showed that contrary to previous knowledge [43] bat fly sex ratios are highly variable among hosts and we demonstrate that this variation is strongly associated with infrapopulation size. Bat flies exhibit strongly male-biased sex ratios in small, and strongly female-biased sex ratios in large infrapopulations, indicating sex-specific aggregation. This finding contradicts predictions of the local mate competition and appears to be rather the result of sex-specific host-preference, mobility or competitive ability. Secondly, we showed that parasites aggregate in larger numbers on female hosts, and parasite abundance increased with increasing host body condition. Given the high transmissibility of bat flies, these results arguably reflect parasite preference for these host traits. Thirdly, we demonstrated that the latter associations are the results of female, but not male bat fly preference for host traits. We argue that this sex-specific preference for host traits is probably the driving force of the density-dependent sex ratio bias across infrapopulations in this host-parasite system.

### Density-dependent sex ratios

Female-skewed sex ratios in small infrapopulations are often described in parasitic communities and are traditionally explained by the local mate competition. Our results refute the latter hypothesis, since small infrapopulations were male-biased, unlike in most parasite communities (e.g. [6, 49]). Moreover, sex ratio approaches unity with increasing infrapopulation sizes in most parasites (e.g. [50]), as large colonies are generally the result of multiple colonisations and are less affected by inbreeding and therefore LMC [6, 49]. On the contrary, here we demonstrate that in large infrapopulations sex ratio shifts to the other extreme, to a strong female bias. To the best of our knowledge, such phenomenon in ectoparasitic communities has never been described before. We believe that such pattern could be of multiple origin, mostly comprising sex-specific traits of the parasites, such as host-preference, competitive ability or mobility, but most likely a combination of these traits. Segregation of the sexes as a result of such sex-specific traits could serve as a driving force shaping the observed density-dependent sex ratio across bat fly infrapopulations.

Our results indicate a stronger preference for host qualities in female compared to male bat flies, presumably partly reflecting nutritional demands. Female bat flies are almost continuously pregnant, nourishing a quickly developing larva within the uterus. Once the larva is fully grown (third-instar) it can take up approximately one third of the total body mass of pregnant females (Tamara Szentiványi, personal observation), while bat fly females had been observed to larviposit one of these at 3 to 9 day intervals [8]. Therefore, reproducing females are expected to have a more intense metabolism and to rely more heavily on permanent and stable resources than males due to their extended parental care [5, 49]. This claim is also supported by the documented higher sensitivity of females to starvation compared to males [1]. As a consequence, females might exert a stronger preference for hosts that are most suitable food resources and can ultimately lead to aggregation of females on certain hosts, especially in systems where free movement of parasites is granted, like in bat flies.

Besides sex-specific host preference, male and female ectoparasites often also differ in their competitive ability. Females are larger than males in most arthropod ectoparasites [51], while this size differences provides females with competitive advantage over males. Sexual size dimorphism is indeed often associated with sex ratio biases, where species with the highest degree of size dimorphism exhibit the strongest bias in their sex ratios [6]. Nonetheless, male and female *N. kolenatii* are similar in lean body size and females only exceed this during pregnancy, but gestation is unlikely to increase their competitive potential. Although female bias in our sample was not significant (*P* = 0.0558), it might indicate a marginally higher mortality or shorter lifespan of males compared to females (e.g. [5, 12, 52]) or it might be indicative of sex-specific competitive potential.

It was also suggested that sex-specific competitive potential is linked to sex-specific dispersal. Generally, the sex subject to more intense competition is more likely to disperse, while no sex-specific bias is expected when local competition equally affects males and females [53]. Mobility or dispersal potential often differs between the sexes in ectoparasites just as much as in birds or mammals [54]. Such sex differences have the potential to result in sex-specific genetic structures, a pattern also documented in the handful ectoparasite species studied to date [4]. Males in bat flies in our study are more likely to disperse than females for a number of reasons. First, small infrapopulations in our sample were extremely male biased, a pattern believed to indicate the higher probability of males to move between hosts and colonise new host individuals. Secondly, dispersion might exert sex-specific costs, especially in spatially heterogeneous environments such as the patchy availability of host individuals. The higher sensitivity to starvation documented in female bat flies might result in their higher philopatry than in males that survive for a longer period of time without a blood meal [1]. How mobility, dispersion potential, competitive potential or host preference vary between the sexes of ectoparasites and how these shape infrapopulation sex ratios need further, experimental attention.

### Host trait preferred by parasitic bat flies

Numerous studies on mammalian-ectoparasite systems describe sex-specific parasitism of hosts, usually demonstrating that males are subject to more intense parasitism than females [27–29, 55]. Contrary to these findings, our results revealed that females of Daubenton’s bats are host of a higher bat fly abundance than males of the same host species. This finding corroborates a number of previous studies showing that female-biased parasitism is more likely to occur in bats than in other mammalian orders (e.g. [14, 24, 32–35, 56]). Such sex differences in host infestation rates might be explained by a range of fundamental differences between the sexes, such as ecology, behaviour and physiology. For instance, female-biased parasitism may be explained by the sex-specific social structure of the hosts. Sexual segregation and the larger roosting colony size of females compared to males is a well-known phenomenon in numerous bat species, including our study species, the Daubenton’s bat [17–20]. Host abundance and physical proximity, the higher temperature at large maternity colonies, the presence of juveniles and their lower behavioural and immunological antiparasite defences were suggested to increase parasite transmission rates, prevalence and abundance [48, 57–59]. Nonetheless, during the mating period male and female Daubenton’s bats tend to aggregate in mixed colonies [20], that is likely to contribute to the horizontal transfer of parasites between female and male hosts. Moreover, the off-host development stages and larviposition of bat flies, as well as their successively shared roosts grant them high transmissibility and colony size has been shown to play no role in the infestation rate of host individuals [38]. Physiological differences between the sexes, such as immunity and hormones were also suggested to influence the defensive ability of hosts against parasites and pathogens. For instance pregnant females tend to suffer from higher parasitism rate than non-reproductive ones [21], while high testosterone levels often induce immunosuppression, which leads to increases parasitism of males [60, 61]. It remains to be explored how host physiology influences host choice in bat flies.

Several other possible factors can play a role in higher parasitism in females, for instance differences in grooming and roosting behaviour. Grooming behaviour is one of the major causes of ectoparasite mortality [48]; however grooming behaviour does not predict the level of parasitism in bats [5]. For instance, great fruit-eating bat (*Artibeus lituratus*) females spend nearly twice as much time with grooming than males during a day [62], still males harbour significantly less ectoparasites [35]. In addition, some authors did not find increased response in grooming behaviour to high infestation by bat flies [63]. Nevertheless, gender differences in grooming behaviour in *M. daubentonii* has not been studied yet. In addition, roost-site selection could also be a predictor of ectoparasite density in bat populations [64].

Sexual size dimorphism may also be a determinant in the level of infestation by parasites in mammals (e.g. [26, 65]); however many authors reported the lack of relationship between host sexual size dimorphism and the level of parasitism in ectoparasites [27, 66]. Differences in size between sexes have been reported in several mammalian species including bats, where the sexual size dimorphism shows a reverse trend, females are the larger sex, especially in the family Vespertilionidae [67–70]. According to Moore & Wilson [26] the parasitism is male biased if sexual size dimorphism appears in favour of males, while female biased if females are the larger sex. Whereas Lindenfors et al. [71] did not observed correlation between the weight and the dimorphism in small mammals (Rodentia, Chiroptera and Insectivora), in our study both sexes of *N. kolenatii* tend to choose females above males and hosts in good body condition. Therefore, our results support the ‘well-fed host strategy’ that states, parasites choose larger (“well-fed”) hosts above the smaller ones (“poorly-fed hosts”) in order to maximize food acquisition [22]. Consequently, besides the factors mentioned above, in our study female-biased host choice may also be the result of the sexual size dimorphism and/or the better health condition of female hosts.

## Conclusions

Biased sex ratios, i.e. the unbalanced number of males and females in a population are highly common in both invertebrate and vertebrate species, including humans. Such sex ratio distortions have important consequences for the social environment and for population demography. Here we explored sex ratio variation in an arthropod ectoparasite and demonstrated that sex ratio shows considerable variance among hosts. Male was the prevalent sex on hosts with few parasites, while females were more prevalent than males on highly infested hosts. Inspecting this sexual segregation, we demonstrated that infra-population sex-ratio is shaped by sex-specific host preference, most likely coupled with sex-specific mobility of the parasites. Our study highlights the importance of sex-specific parasite traits in the development of population-wide sex-ratio distortions and the importance to account for parasite sex in parasitological studies.

## Additional file

Additional file 1: Table S1. Data for all collected host and parasite individuals (location, date, host and parasite traits are included). (XLSX 18 kb)
